# Socio-economic development and emotion-health connection revisited: a multilevel modeling analysis using data from 162 counties in China

**DOI:** 10.1186/s12889-016-2926-z

**Published:** 2016-03-12

**Authors:** Zonghuo Yu, Fei Wang

**Affiliations:** Jiangxi Normal University, Room 313, Jieqiong Buliding, No. 99, Ziyang Road, Nanchang, China; Jiangxi Key Laboratory for Psychology and Cognitive Science, Nanchang, China; Tsinghua University, Beijing, China

**Keywords:** GDP, Emotion-health connection, Accessibility of medical resources, Educational status

## Abstract

**Background:**

Substantial research has shown that emotions play a critical role in physical health. However, most of these studies were conducted in industrialized countries, and it is still an open question whether the emotion-health connection is a “first-world problem”.

**Methods:**

In the current study, we examined socio-economic development’s influence on emotion-health connection by performing multilevel-modeling analysis in a dataset of 33,600 individuals from 162 counties in China.

**Results:**

Results showed that both positive emotions and negative emotions predicted level of physical health and regional Gross Domestic Product Per Capita (GDPPC) had some impact on the association between emotion and health through accessibility of medical resources and educational status. But these impacts were suppressed, and the total effects of GDPPC on emotion-health connections were not significant.

**Conclusions:**

These results support the universality of emotion-health connection across levels of GDPPC and provide new insight into how socio-economic development might affect these connections.

## Background

A great deal of evidence has suggested that emotions play a critical role in physical health, such that negative emotions (NE) are detrimental while positive emotions (PE) are beneficial [1–5]. One caveat in this literature, however, is that most of the existing studies were conducted in industrialized countries. As suggested by SD Pressman, MW Gallagher and SJ Lopez [6], people in developed countries have met the more basic needs such as safety and physiological needs and focus more on their emotional well-being than those in developing countries. To test whether the emotion-health connection is indeed a “first-world problem”, they analyzed a dataset of 150,048 individuals from 142 countries. The results does not support their original speculation, indicating that both NE and PE could independently and significantly predict physical health regardless of countries’ Gross Domestic Product (GDP) per capita, even after controlling for fulfillments of basic need. However, PE-health link was found to be stronger in countries with lower GDP per capita, suggesting that a country’s level of development might have some impact on the emotion-health connection.

Although Pressman et al.’s study has provided valuable insight into socio-economic development’s influence on emotion-health connection, its approach of cross-national comparison leaves it vulnerable to confounding factors. For example, countries’ level of socio-economic development is correlated with culture [7–9]. While some researchers have found weaker emotion-health connection in Eastern cultures [10–12], others have found the reverse pattern [13]. Even though there is no consensus regarding culture’s role in the emotion-health relationship, it could potentially hinder testing the moderating effect of development level. A proper test for the “first-world problem” hypothesis requires these confounding factors to be controlled for.

### Aim of this study

In the study reported here, we used a representative dataset collected in China to examine whether socio-economic development (indexed by regional GDP per capita) affects the strength of the emotion-health connection. Using China as a testing ground for the “first-world problem” hypothesis offers several advantages. Firstly, China is more ethnically and politically unified than other regions in the world (e.g. such as Europe or America) with more than 90 % of its population belongs to Han nationality [14]; using data from a single ethnicity naturally controls for many confounding variables (e.g., language, culture) inherent in previous cross-national comparisons. Secondly, development in China is uneven across regions, and levels of regional GDPs cover both ends of the spectrum from “third-world” to “first-world”. For example, the GDP per capita ranges from Gui Zhou Province’s CNY 13,119 (≈ USD 2,112) to Beijing’s CNY 73,105 (≈ USD 11,890) among provinces in China in 2010 [15]. Such a wide range of level of development, combining with ethnical, political and cultural homogeneity, makes China an ideal place to test whether and how emotion-health connection is moderated by socio- economic development.

Besides testing the “first-world problem” hypothesis, we looked further into some possible mechanisms that may account for the effect of economic development on the linkage between emotion and health. SD Pressman, MW Gallagher and SJ Lopez [6] interpreted the weaker PE-health connection in developed countries as medical interventions downgrading the impact of emotion on health. In the current study, we tested whether accessibility of medical resources could explain GDP’s effect on the emotion-health link. Furthermore, socio-economic development is linked with educational status, which could facilitate emotion-regulation ability [16], thus also weakening the association between emotion and health.

## Methods

### Participants

The data used in this study was collected in 2010 by the Institute of Social Science Survey of Peking University(extensive information about the survey can be found at www.isss.edu.cn/cfps/) for the China Family Panel Studies (CFPS) which focused on the economic, as well as the non-economic, wellbeing of the Chinese population, and promised to provide the most comprehensive and highest-quality survey data in contemporary China [17–19]. And we received permission from the Institute of Social Science Survey of Peking University to use it.

Participants were 33,600 individuals (17,314 females, 16,286 males; age ranging from16 to 110 years old, M = 45.51, SD = 16.41) from 162 counties of 25 provinces in China. The CFPS sample was obtained through a stratified multi-stage sampling procedure to represent 95 % of the total Chinese population in 2010 (for details, see: [20]). The mean size of the county samples was 207.41 (range = 43 – 443). 30,763 participants self-identified as ethnic Han Chinese, 2756 as non-Han minorities, and 81 respondents did not report their ethnic. In order to exclude the effect of culture, we analyzed only Han Chinese [14].

### Ethical considerations

The data used in this article were supported by the project named China Family Panel Studies (CFPS), which was implemented by Institute of Social Science Survey, Peking University. All participants have given detailing information about CFPS and willing to take part in.

### Measure

#### Positive emotion

PE was measured with a single self-report five-point happiness item enquiring whether individuals were happy. Previous studies have found evidence pertaining to the validity of such single-item measure [21].

#### Negative emotion

NE was measured with six items asking how often one felt depressed, agitated or upset, nervous, hopeless about future, felt that everything was difficult or thought life was meaningless in the past month. Participants answered the questions using a 5-point scale: 1 = *Almost every day*, 2 = *2*-*3 Times a week*, 3 = *2*-*3 Times a month*, 4 = *Once a month*, 5 = *Never*. Cronbach's Alpha of these six items is 0.932.

#### Health

Self-reported health was assessed with one question regarding self-perceived health status using a 5-point scale: 1 = *Healthy*, 2 = *Fair*, 3 = *Relative unhealthy*, 4 = *Unhealthy*, 5 = *Extremely unhealthy*. Responses were reverse-recoded so that higher scores indicated superior health.

#### Socio-economic development

Gross domestic product Per capita (GDPPC) was used as an indicator of socio-economic development. The data of GDPPC was provided by the China Family Panel Studies, and the levels of GDPPC across 162 counties range from CNY 3,191(≈ USD 519) to CNY 320,026(≈ USD 52,068).

#### Educational status

Educational status was measured as participant’s total years of education.

#### Accessibility of medical resources

The extent to which medical resources was accessible was measured reversely by the travel time to nearest health facility.

#### Controlling variables

The following variables were added into the model as covariates:

#### Gender

Participants’ gender was coded as 1 = *Male*, 0 = *Female*.

#### Age

Participants reported their birthday, and age in years was calculated.

#### Health behavior

Two items measured whether participants have ever drunk or smoked.

#### Body-Mass Index

Participants reported their height and weight. Body-Mass Index was calculated, and two dummy variables were created for BMI ≥ 25 and BMI < 18.

#### Family size

Participants reported how many people besides themselves were living together in their family.

#### Job status

Participants’ job status was classified into five categories: working, doing housework, retired, rich and do not need to work, and unemployed. Four dummy variables were created using unemployed as the baseline.

#### Income

Income was assessed with total personal income last year and family income per capita.

#### Marriage status

Participants’ marriage status was classified into three categories: married, divorced or widowed, and unmarried. Two dummy variables were created using unmarried as the baseline.

### Data analysis

The relationships between GDP and emotion-health connection were analyzed in two steps. First, we computed the correlation coefficient of PE/NE and self-reported physical health within each of the 162 counties in China and computed the summary effect of these correlations. Second, we used a multilevel random-coefficient model [22] to test whether and how GDPPC moderated the association between physical health and emotions. In this model, positive emotion, negative emotion, years of education, travel time to the nearest health facility and controlling variables were placed in the individual-level regression model as predictors. It is worth noting that GDPPC’s moderating effects on the emotion-health connection are cross-level interactions; previous research has shown that for this type of effect, a minimum of 30 groups and 30 observations within each group is required to achieve sufficient power [23]. In the current study, the number of groups was 162, and the mean number of observations per group was 207.41, both of which were greatly exceeding the above recommendation levels, ensuring that the tests were of adequate power.

In order to test the mechanism of how GDPPC moderated the emotions-physical health connection, educational status and accessibility of medical resources were also added into county-level slope model to predict PE-health and NE-health connection, and Sobel test was carried out to assess the indirect effects of GDPPC on emotion-health connection through the mediators. Recent development in mediation analysis suggests that indirect effect of X on Y through M can be directly tested in absence of a statistically significant total effect of X on Y [24], therefore we tested GDPPC’s indirect effects on emotion-health connections through accessibility of medical resources and educational status regardless of the total effects’ significance. Individual income, family income per capita (FIPC) and GDPPC are logarithmically transformed (e.g., [25]) and all non-dichotomous predictors were grand-mean-centered and entered as random slopes.

## Results

### Regional emotion-health correlations

Among 162 counties in China, PE was a strong positive predictor of health in 112 counties (59 counties at *p* < .01 level and 53 counties at *p* < .05 level), and NE was a strong negative predictor of health in 144 counties (139 counties at *p* < .01 level and 5 counties at *p* < .05 level). In the random effect analysis of the overall effect size, we found a significant positive correlation between PE and physical health (*r* = .202, 95 % CI [.19, .22], Z = 25.94, *p* < .001) and a significant negative correlation between NE and physical health(*r* = −.3458, 95 % CI [−.36, −.33], Z = −35.52, *p* < .001). Test of heterogeneity suggested that both PE-health connection and NE-health connection significantly varied from county to county (Q = 325.08, df = 161, *p* < .001; Q = 620.34, df = 161, *p* < .001, respectively).

### Moderating effect of GDP per capita on emotion-health correlation

At individual-level, positive emotion, education status, individual income, family income per capita and family size were significantly positive predictors of physical health, while age, unhealthy behaviors were significantly negative predictors of physical health. It was also found that BMI, job status and marriage status were significant predictors of physical health (see Table 1 for details).

**Table 1:** Estimation of health’s multilevel model

	Effects	Estimate	S.E.	Est./S.E.	95 % CI	*P*-value
Intercept of health	intercept	3.952	0.041	96.680	[3.872, 4.032]	0.000
	GDPPC coefficient	−0.117	0.065	−1.783	[−0.245, 0.012]	0.075
Slope of PE	intercept	0.089	0.006	14.013	[0.077, 0.102]	0.000
	GDPPC coefficient	0.019	0.016	1.241	[−0.011,0.050]	0.215
	AMR coefficient	0.002	0.001	3.055	[0.001, 0.003]	0.002
	ES coefficient	−0.004	0.002	−2.326	[−0.008, 0.001]	0.020
Slope of NE	intercept	−0.073	0.003	−26.988	[−0.078, 0.068]	0.000
	GDPPC	0.002	0.009	0.274	[−0.015, 0.019]	0.784
	AMR coefficient	−0.001	0.001	−1.608	[−0.002, 0.000]	0.108
	ES coefficient	0.004	0.002	2.014	[0.000, 0.007]	0.044
Slope of gender	intercept	0.145	0.013	10.951	[0.119, 0.171]	0.000
Slope of age	intercept	−0.014	0.001	−25.528	[−0.015,-0.013]	0.000
Slope of education	intercept	0.006	0.002	3.894	[0.003, 0.009]	0.000
Slope of AMR (individual-level)	intercept	0.001	0.001	2.612	[0.001,0.002]	0.009
Slope of income	intercept	0.058	0.011	5.282	[0.036,0.079]	0.000
Slope of FIPER	intercept	0.097	0.017	5.546	[0.062, 0.131]	0.000
Slope of family size	intercept	0.017	0.004	4.652	[0.010, 0.025]	0.000
Slope of having job	intercept	0.121	0.015	8.231	[0.092, 0.150]	0.000
Slope of Retried	intercept	0.054	0.059	0.916	[−0.061, 0.169]	0.359
Slope of housework	intercept	0.177	0.026	6.696	[0.125, 0.229]	0.000
Slope of Rich, at home	intercept	0.201	0.069	2.897	[0.065, 0.337]	0.004
Slope of drink	intercept	−0.255	0.028	−9.281	[−0.309, 0.202]	0.000
Slope of Smoking	intercept	−0.173	0.024	−7.144	[−0.221, 0.126]	0.000
BMI ≥ 25	intercept	−0.045	0.015	−2.972	[−0.075, 0.015]	0.003
BMI < 18	intercept	−0.209	0.023	−9.126	[−0.254, 0.164]	0.000
Slope of married	intercept	−0.094	0.023	−4.017	[−0.140, 0.048]	0.000
Slope of widowed or divorced	intercept	0.059	0.035	1.681	[−0.010, 0.127]	0.093
AMR at county-level	intercept	−0.233	0.430	−0.541	[−1.077, 0.611]	0.588
	GDPPC coefficient	5.367	0.976	5.499	[3.454, 7.280]	0.000
ES at county-level	intercept	0.073	0.131	0.593	[−0.179, 0.335]	0.553
	GDPPC coefficient	3.577	0.298	12.017	[2.994, 4.160]	0.000

*AMR* accessibility of medical resources, *GDPPC* GDP per capita, *ES* educational status, all non-dichotomous predictors were grand-mean-centered

At county-level, GDPPC was a significant negative predictor of the intercept of physical health (γ = −0.148, SE = 0.066, 95 % CI [−0.277, −0.020], *p* = 0.024). Critically, the direct effect of GDPPC on PE-health relationship did not significant (γ = 0.015, SE = 0.014, 95 % CI [−0.013, 0.044], *p* > .250), nor NE-health relationship (γ = 0.010, SE = 0.006, 95 % CI [−0.003, 0.022], *p* = .120).

### Mediating roles of accessibility of medical resources and educational status

**Fig. 1:**
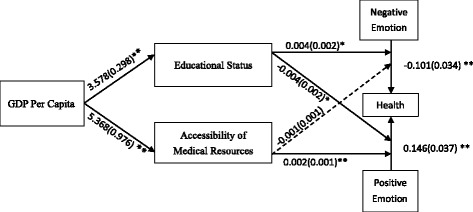
Mediation model showing the relationships between GDPPC and emotion-health connection as mediated by accessibility of medical resources and educational status. In this multilevel random-coefficient model, positive emotion, negative emotion, and health were individual-level variables, and GDPPC, educational status and accessibility of medical resources were county-level variables. Unstandardized regression coefficients are shown, and standard errors are given in parentheses. One asterisk indicate significant coefficients (*p* < .05), two asterisks indicate significant coefficients (*p* < .01)

As shown in Fig. 1, GDPPC was a significant predictor of years of education and accessibility of medical resources (γ = 3.577, SE = .298, 95 % CI [2.994, 4.160], *p* = .000; γ = 5.367, SE = .976, 95 % CI [3.454, 7.280], *p* < .001, respectively). Adding these two variables in county-level slope model of GDP-emotion-health yielded that NE-health connection was significantly affected by years of education (γ = .004, SE = .002, 95 % CI [.000, .006], *p* = .044) but not by accessibility of medical resources (γ = −.001, SE = .001, 95 % CI [−.002, .000], *p* = .110). PE-health connection, on the other hand, was significantly affected by both educational status (γ = −.004, SE = .002, 95 % CI [−.008, −.001], *p* = .020) and accessibility of medical resources (γ = .002, SE = .001, 95 % CI [.001, .003], *p* = .002). Analysis of residual variances showed that NE-health wasn’t fully explained (ε = 0.001, SE = 0.000, 95 % CI [.000, .001], *p* < .001), while PE-health was (ε = 0.000, SE = 0.000, 95 % CI [.000, .000], *p* > .250).

To exam whether educational status and medical resources are indeed mediating the relationship between GDPPC and emotions-physical health, we carried out Sobel test on the indirect effects. For educational status, both the indirect effects of GDPPC on PE-health connection through years of education and GDPPC on NE-health connection through years of education were significant (γ = −.015, SE = .007, 95 % CI [−.028, −.002], *p* = .022, and γ = .013, SE = .006, 95 % CI [.000, .025], *p* = .047, respectively). For accessibility of medical resources, the indirect effect of GDPPC on PE-health connection through accessibility of medical resources was significant (γ = 0.009, SE = 0.003, 95 % CI [.002, .016], *p* = .008), while the indirect effect of GDPPC on NE-health connection through accessibility of medical resources was not (γ = −0.004, SE = 0.003, 95 % CI [−.010, .001], *p* = .123).

Educational status and accessibility of medical resources fully mediated the effects of GDPPC on emotions-health connections such that GDPPC’s direct effects were still non-significant after adding the two variables (γ = 0.019, SE = 0.016, 95 % CI [−.011, .050], *p* = .215, and γ = 0.002, SE = 0.009, 95 % CI [−.015, .019], *p* > .250). Furthermore, neither the total effect of GDPPC on PE-health relationship nor the total effect of GDPPC on NE-health relationship was significant (γ = 0.076, SE = 0.046, 95 % CI [−.015, .167], *p* = .100, and γ = 0.015, SE = 0.022, 95 % CI [−.028, .058], *p* > .250, respectively).

## Discussion

Using a large-scale dataset collected from a representative sample in China, we replicated Pressman et al.’s [6] finding that both PE and NE could independently and significantly predict self-reported health, once again showing that emotions play a critical role in physical health. Contrary to their results, however, neither PE-health connection nor NE-health connection was moderated by regional GDPPC. Given that our data was collected within a single nation, which naturally controlled for many confounding factors such as culture [6, 10, 26], the current study provided a more rigorous test of socio-economic development’s effect on the association between emotion and health, and suggested that the emotion-health connection is not, after all a ‘first-world problem’.

Despite of the non-significance of GDPPC’s total effects on emotion-health connections, several mediating pathways through accessibility of medical resources and educational status were confirmed. To interpret GDPPC’s moderation effect on PE-health connection in their results, SD Pressman, MW Gallagher and SJ Lopez [6] proposed that in developed regions medical interventions downgraded the impact of emotions on health. Our empirical test of the pathway showed that it was statistically significant, but in opposite direction: GDPPC predicted easier accessibility of medical resources, which actually strengthened PE-health connection. One interpretation is that positive emotion might promote people to utilize available medical resources to deal with physical problem. In addition, educational status showed a significant mediating effect between GDPPC and PE-health, such that higher GDPPC was linked with higher educational status, which in turn predicted weaker association between PE and health. Previous studies have shown that education could facilitate emotion-regulation ability [e.g., 16], which might in turn downgrades emotions’ impact on health. This interpretation is also applicable to NE-health connection, which shifted closer to zero as educational status increased. Overall, these results provide new insight into the underlying mechanism through which socio-economic development might affect the emotion-health connection. Beyond the two mediators examined here, other pathways in opposite direction may also exist, rendering the total effects of socio-economic development on emotion-health connections to be non-significant, which is worthy of future research. Furthermore, different from the results in previous study [6], the study shows that it’s equally important for them to alter their levels of happiness or sadness as the riches. So, policy makers must remind themselves of creating conditions to meet not only the rich's spiritual pursuit, but also the poor’s.

One limitation of the current study is that several key variables, such as PE and health status, were assessed with single-item measures. Theoretically, these constructs are multidimensional and have different sub-components, and there are multi-item scales available [27, 28]. In addition, personal health could also be assessed with more objective measures such as illness status. Unfortunately, the CFPS only contains the self-rated single-item ones. Nonetheless, previous research has shown that even these general self-rated single-item measures still demonstrated adequate predictive validity of key outcome variables [29]. Furthermore, although using a sample from single country rules out many potential confounding factors, it also leaves one to speculate whether characteristics of the specific country could affect the results and whether they can be generalized to other countries. For example, previous cultural-psychological studies showed that in Western cultures, emotions are the reflection of the inner world, while in East Asian cultures they are more affected by the social relationships and interpreted as products of situation [10, 26, 30, 31], and such different approaches towards emotion may in turn affect the emotion-health connection. However, existing studies on cultural differences are limited in number and yielded mixed results [e.g., 10, 11–13]. In the future, it’s necessary to measure culture variables directly and examine their impact on the emotion-health connection.

## Conclusion

Although GDPPC had some impact on emotion-health connection through accessibility of medical resources and educational status, the total moderating effects were not significant, supporting that emotional influences on personal health are not a “first-world problem”, but universal across levels of socio-economic development.
